# Hyperthermia and associated changes in membrane fluidity potentiate P2X7 activation to promote tumor cell death

**DOI:** 10.18632/oncotarget.18595

**Published:** 2017-06-21

**Authors:** Paola de Andrade Mello, Shu Bian, Luiz Eduardo Baggio Savio, Haohai Zhang, Jingping Zhang, Wolfgang Junger, Márcia Rosângela Wink, Guido Lenz, Andréia Buffon, Yan Wu, Simon Christopher Robson

**Affiliations:** ^1^ Laboratório de Análises Bioquímicas e Citológicas, Faculdade de Farmácia, Universidade Federal do Rio Grande do Sul (UFRGS), Porto Alegre, RS, Brazil; ^2^ Department of Medicine, Beth Israel Deaconess Medical Center, Harvard Medical School, Harvard University, Boston, MA, USA; ^3^ Department of Gastroenterology, Tianjin Union Medical Center, Tianjin, P.R. China; ^4^ Programa de Imunobiologia, Instituto de Biofísica Carlos Chagas Filho, Universidade Federal do Rio de Janeiro, Rio de Janeiro, RJ, Brazil; ^5^ Department of Liver Surgery, Peking Union Medical College Hospital, Chinese Academy of Medical Sciences and Peking Union Medical College, Beijing, P.R. China; ^6^ Department of Surgery, Beth Israel Deaconess Medical Center, Harvard Medical School, Harvard University, Boston, MA, USA; ^7^ Laboratório de Biologia Celular, Universidade Federal de Ciências da Saúde de Porto Alegre (UFCSPA), Porto Alegre, RS, Brazil; ^8^ Departamento de Biofísica e Centro de Biotecnologia, Universidade Federal do Rio Grande do Sul (UFRGS), Porto Alegre, RS, Brazil

**Keywords:** purinergic signaling, hyperthermia, membrane fluidity, cancer therapy, colon cancer

## Abstract

Extracellular ATP (eATP) accumulation within the tumor microenvironment (TME) has the potential to activate purinergic signaling. The eATP evoked signaling effects bolster antitumor immune responses while exerting direct cytotoxicity on tumor cells and vascular endothelial cells, mediated at least in part through P2X7 receptors. Approaches to augment purinergic signaling in TME e.g. by ectonucleotidase CD39 blockade, and/or boosting P2X7 functional responses, might be used as immunomodulatory therapies in cancer treatment. In this study, we delineated the translatable strategy of hyperthermia to demonstrate impacts on P2X7 responsiveness to eATP. Hyperthermia (40°C) was noted to enhance eATP-mediated cytotoxicity on MCA38 colon cancer cells. Increased membrane fluidity induced by hyperthermia boosted P2X7 functionality, potentiating pore opening and modulating downstream AKT/PRAS40/mTOR signaling events. When combined with cisplatin or mitomycin C, hyperthermia and eATP together markedly potentiate cancer cell death. Our data indicate that clinically tolerable hyperthermia with modulated P2X7-purinergic signaling will boost efficacy of conventional cancer treatments.

## INTRODUCTION

Adenosine triphosphate (ATP) is well known to provide the intracellular energy currency of cells, but is also present at low levels in the extracellular space under physiological conditions. Higher levels of extracellular ATP are perceived as a danger signal by cells in pathological processes [1]. The presence of high levels of extracellular ATP has been noted in the tumor microenvironment [2, 3]. Such heightened pericellular ATP levels have been demonstrated to increase tumor cell death through multiple mechanisms inclusive of: augmentation of antitumor immunity, direct induction of tumor cell death, and blockade of tumor angiogenesis [4, 5].

Although antitumor actions of extracellular ATP may occur through activation of several type 2-purinergic receptors (P2R), P2X7 is thus far the most important ATP-receptor [1, 5]. P2X7 is the only P2X receptor capable to form a membrane pore permeable to large molecules (up to 900 Da), resulting in tumor cell apoptosis [6].

Low levels of extracellular ATP that develop as a consequence of scavenging, or defective P2X7 activation per se, have been linked to immunosuppressive responses. These provoke cancer development by allowing tumor cells to escape from the P2X7 controlled pro-apoptotic mechanisms [7, 8]. Curiously, basal or low level activation of P2X7 receptor promotes tumor cell growth [9]. A therapeutic modality that increases P2X7 tumoricidal functions, thereby preventing cancer cell escape, would be an important advance.

We have recently highlighted the modulation of P2X7 receptor signaling in induction of cancer cell death and proposed the use of purine-related drugs as adjunctive agents in cancer therapy [4]. We have demonstrated that pulsed treatment of MCA38 colon cancer cells with high levels of ATP was able to induce cancer cell death, exclusively via P2X7 [4]. We have further delineated PI3K/AKT and AMPK-PRAS40-mTOR as the downstream pathways initiated by ATP-P2X7 signals. These two signaling axes act synergistically to elicit maximal levels of tumor cell death by disrupting homeostatic cell growth and the process of autophagy.

More recently, we have also shown that high levels of extracellular ATP promote human cervical cancer cell death through a joint mechanism linked by both P2X7 activation and associated adenosine formation [10]. Cells expressing high levels of P2X7 are susceptible to ATP-P2X7 cytotoxicity, whereas the death of cells with low P2X7 levels appeared to be caused by adenosine uptake and subsequent AMPK phosphorylation, dATP accumulation, p53 activation, as well as autophagy induction [10].

Interestingly, pore-forming activity of P2X7 has been linked to remodeling of membrane lipid rafts viz. the rate of agonist-evoked pore formation is enhanced by cholesterol depletion using methyl-β-cyclodextrin (MCD) whereas is inhibited by cholesterol loading [11]. Moreover, lipid raft remodeling can be induced by heat stress, non-proteotoxic membrane lipid-interacting chemicals, such as Bimoclomol, or the membrane fluidizer benzyl alcohol (BA) [12, 13]. These so-called “membrane-lipid therapy” promote changes in the nanostructure and physicochemical state of plasma membranes by interfering with protein and lipid interactions, thereby altering cell transmembrane signals [13]. However, whether such membrane-lipid therapies impact P2X7 functionality, and to what extent this is feasible, remain elusive.

Here, we demonstrate that mild heat stress (or hyperthermia; 40°C) augments ATP agonist efficacy thereby stimulating P2X7 pore opening and dilatation. Ultimately, this leads to dramatically enhanced MCA38 colon cancer cell death, as mediated through AKT/PRAS40/mTOR signaling. Hyperthermia-induced increases in membrane fluidity facilitate P2X7 pore formation and promote cell death. Furthermore, we have also shown that cisplatin or mitomycin C in combination with hyperthermia and extracellular ATP is more effective in inducing cancer cell death. Our results suggest the potential utility of P2X7 hyperactivation by hyperthermia as an adjunct therapy in the treatment of cancer.

## RESULTS

### Hyperthermia increases ATP tumoricidal activity via P2X7-AKT/PRAS40/mTOR signaling

**Figure 1:**
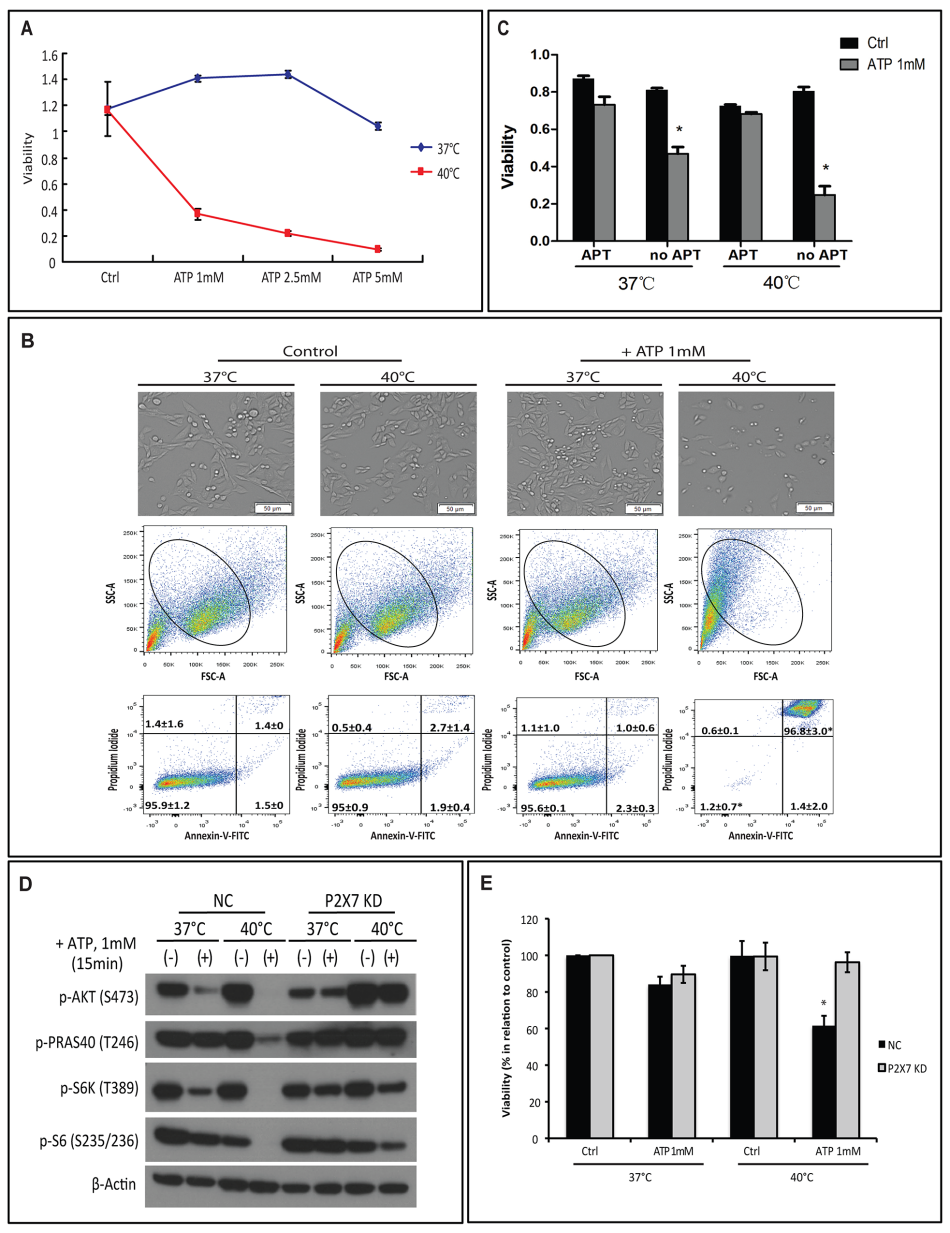
Hyperthermia potentiates ATP cytotoxicity in a P2X7-dependent manner **(A-B)** MCA38 wild type (WT) cells were treated with exogenous ATP at indicated concentrations and temperatures for 15 min. Cells treated with media served as control (Ctrl). Cell viability was determined using CCK-8 **(A)** and images of live cells were captured by Celigo (upper) and cell apoptosis/necrosis was evaluated by FACS (middle and bottom) **(B)**. **(C)** Cells were left untreated or pre-treated with 2.5 μg/ml of APT102 (soluble ectonucleotidase CD39) for 30 min before exposed to ATP 1 mM for 30 min at indicated temperatures. Cell viability was measured 24 hr later. **(D-E)** MCA38 P2X7 KD (P2X7-deficient) and NC (negative control) cells were treated with ATP (1 mM for 15 min) at 37°C or 40°C. AKT/PRAS40/mTOR signaling pathway was analyzed by Western blotting **(D)** and cell viability was evaluated after 24 hr **(E)**. *p < 0.05 as compared to control (one-way ANOVA, followed by Tukey pos-test). Bars, 50 μM.

We have previously shown that, at 37°C, 1 mM ATP will rapidly cause cytotoxicity in MCA38 colon cancer cells at 30 min [4]. Herein, we have investigated whether hyperthermia would sensitize cancer cells to ATP cytotoxicity. We first titrated growth responses of wild type (WT) MCA38 cells to increased ATP levels (ranging from 1, 2.5 and 5 mM) for 15 min at 37°C, 40°C or 42°C. As shown in Supplementary Figure 1, 42°C treatment was capable to promote cancer cell death per se. ATP cytotoxicity was selectively and dramatically enhanced by mild heat stress (hyperthermia; 40°C) in a dose-dependent manner (Figure 1A). In contrast, at 37°C, cytotoxicity was only noted with much higher concentration of ATP (5 mM). Therefore, 1 mM of ATP for 15 min and the temperature of 40°C were chosen for the hyperthermia experimentation, unless otherwise stated. We observed that elevation of cytotoxicity induced by combination of ATP and hyperthermia was associated with increased cell death (Figure 1B; upper panel) and cell shrinkage (Figure 1B; middle panel). We also found that ATP/hyperthermia-evoked cell death has both apoptotic and necrotic characteristics (Figure 1B; bottom panel). Moreover, such antitumor activity was near completely counteracted by co-treatment with APT102, a soluble ectonucleotidase, when cells were exposed to ATP (1 mM) for 30 min, either at 37°C or 40°C (Figure 1C), suggesting the exclusive involvement of ATP in antitumor actions under these experimental settings.

We established previously that ATP induces MCA38 cancer cell death through two P2X7-mediated intracellular signaling networks: PI3K/AKT and AMPK-PRAS40-mTOR [4]. We next examined whether there was an exclusive link between hyperthermia and P2X7 signaling by employing the P2X7-deficient MCA38 cell line (P2X7 KD) [4].

We first confirmed resistance of P2X7 KD cells to cytotoxicity induced by ATP and hyperthermia. In the negative control cell line (NC), as linked to cell death, we noted additive effects of hyperthermia on the two ATP-P2X7 signaling axes: 40°C treatment decreased phosphorylation of both AKT and mTOR pathway components including PRAS40, S6K and S6 (Figure 1D and 1E). These effects were not seen in the P2X7 KD cell line (Figure 1D and 1E).

### Hyperthermia facilitates ATP cytotoxicity by increasing P2X7 functionality

**Figure 2:**
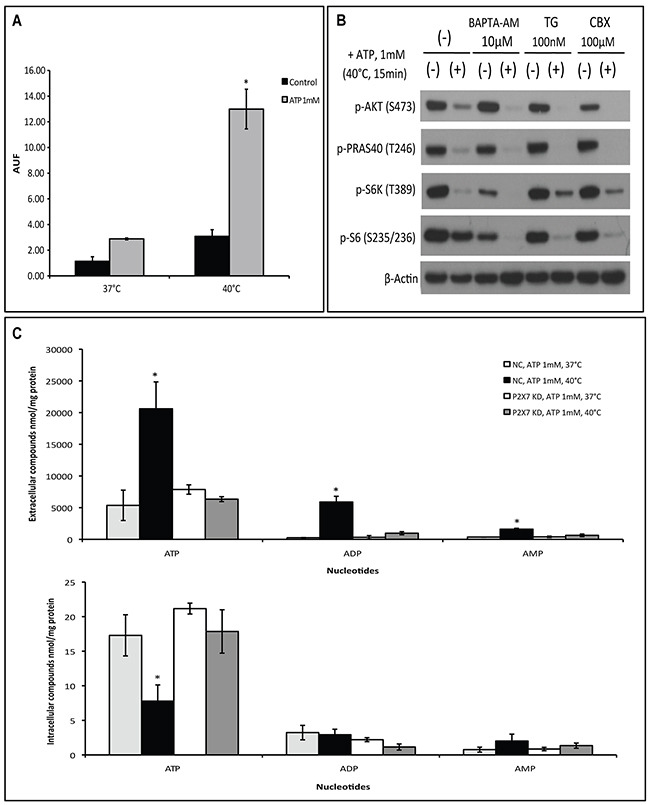
Hyperthermia increases ATP-tumor killing activity by enhancing P2X7 pore formation independently of Ca^2+^ influx and pannexin/connexin interaction **(A)** P2X7 functionality upon ATP/hyperthermia treatment measured by etidium bromide (EtBr) uptake. Cells were left untreated or treated with ATP (1 mM) for 15 min at 37°C or 40°C, followed by whole cell fluorescence measurement (AUF), as described in Material and Methods. **(B)** Cells were pre-incubated with BAPTA-AM, thapsigargin (TG) or Carbenoxolone (CBX) prior to heat-ATP pulse treatment, followed by western blot analysis of AKT/PRAS40/mTOR signaling pathway. Cells treated with media served as control. **(C)** NC (negative control) and P2X7 KD (P2X7-deficient) cells were exposed to ATP for 15 min at 37°C or 40°C and extracellular and intracellular adenine nucleotide levels were determined by HPLC. *p < 0.05 in contrast to control (one-way ANOVA, followed by Tukey pos-test, mean ± SD).

Next, we evaluated the impact of hyperthermia on P2X7 functionality including pore formation capacity, Ca^2+^ influx, and associations with pannexin or connexin channels. First, ethidium bromide uptake assay was performed to measure the membrane pore-opening activity of P2X7 in a non-selective manner. We observed that ATP markedly stimulates ethidium bromide uptake when cells were incubated at 40°C (Figure 2A).

Second, two chemical compounds that differently alter cytosolic Ca^2+^ levels: BAPTA-AM, a selective cell permeable calcium chelator that depletes Ca^2+^ intracellular stores, and thapsigargin (TG), a potent inhibitor of endoplasmic reticulum Ca^2+^-ATPase causing an immediate increase in cytoplasmic Ca^2+^ levels, were employed to examine changes in the ATP-P2X7 intracellular signaling pathway upon hyperthermia treatment. As shown in Figure 2B, neither BAPTA-AM nor TG was able to block hyperthermia-induced ATP-P2X7-AKT/PRAS40/mTOR signaling transduction. Of note, prevention of extracellular Ca^2+^ influx with EDTA also fails to counteract ATP cytotoxicity at 40°C (Supplementary Figure 2). Moreover, carbenoxolone (CBX), a pharmacological inhibitor of pannexin/connexin channels that are often linked to P2X7 activation, also failed to block the hyperthermia-associated changes to the ATP-P2X7 signaling cascade (Figure 2B).

It is known that non-selective pore opening upon P2X7 activation facilitates cytosolic adenine nucleotide efflux into the extracellular space [4]. We then assessed levels of both extracellular and intracellular adenine nucleotides in our experimental settings by HPLC. We found that co-treatment of ATP with hyperthermia significantly increased levels of extracellular adenine nucleotides (inclusive of ATP, ADP and AMP), accompanied with concomitant decreases in intracellular levels of ATP (Figure 2C). Such phenomena were not seen in cells deficient of P2X7 (Figure 2C). Of note, basal levels of extracellular nucleotides were not altered by the changes in temperatures *(data not shown)*.

These data suggest that hyperthermia/ATP-evoked pore formation is an intrinsic property of P2X7 receptor that is independent of calcium signaling and pannexin/connexin channels.

### Hyperthermia-induced P2X7 sensitivity to ATP is not associated with lipid raft cholesterol content

It has been demonstrated that membrane interaction of P2X7 with lipid rafts regulates the receptor channel properties [11], and heat stress perturbs lipid raft integrity [12, 13]. As such, we next evaluated the role of cholesterol within lipid rafts in hyperthermia and ATP-mediated hyperactivation of P2X7.

**Figure 3:**
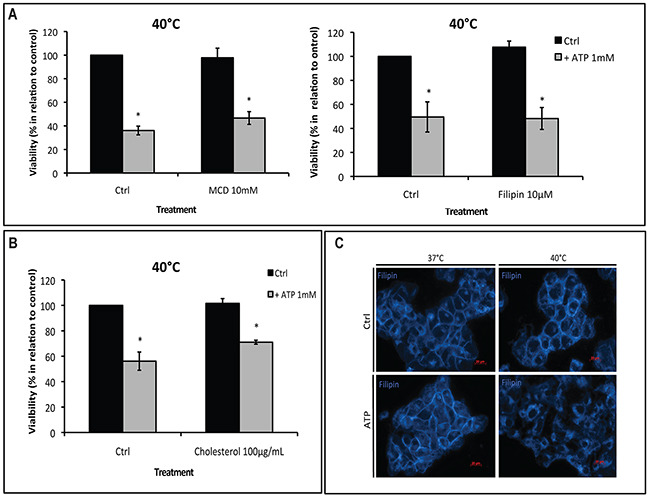
Hyperthermia-increased P2X7 functionality is independent of lipid rafts **(A-B)** MCA38 cells were treated with methyl β-cyclodextrin (MCD) (left) or filipin (right) **(A)** or cholesterol **(B)**, before being pulse treated with ATP (1 mM) for 15 min at 40°C. 24 hr later, cell viability was evaluated. Cells treated with media served as control (Ctrl). **(C)** Cholesterol staining with filipin showing cholesterol rearrangement at the plasma membrane after treatment with hyperthermia and ATP. *p < 0.05 in contrast with control (two-way ANOVA, followed by Bonferroni pos-test, mean ± SD). Bars, 20 μM.

First, we examined the involvement of cholesterol, an essential component of plasma membrane lipid rafts, in hyperthermia/ATP-induced cytotoxicity. Cells were pre-treated with two chemical cholesterol disruptors: methyl β-cyclodextrin (MCD), which depletes lipid rafts cholesterol or filipin, which is highly fluorescent and binds specifically to cholesterol, before cells were exposed to ATP and/or hyperthermia. In Supplementary Figure 3A, we show that filipin forms a multimeric globular complex with membrane cholesterol, whereas MCD (10 mM) effectively depletes rafts of cholesterol. We observed that MCD or filipin had no effects on ATP cytotoxicity at 40°C (Figure 3A).

Alternatively, stabilization of lipid rafts by loading cells with soluble cholesterol also failed to alter ATP tumor-killing activity (Figure 3B), indicating that cholesterol content within lipid rafts is not responsible for controlling P2X7 functionality. In addition, immunoprecipitation assays suggested there were no physical interactions between P2X7 and the two lipid raft markers caveolin-1 or flotillin-2 (Supplementary Figure 3B).

However, interestingly, combination of hyperthermia and ATP indeed led to reorganization of membrane cholesterol (Figure 3C), indicating that changes in the plasma membrane structure per se may modulate P2X7 function.

### Hyperthermia-induced membrane fluidity is responsible for enhanced P2X7 sensitivity to ATP

**Figure 4:**
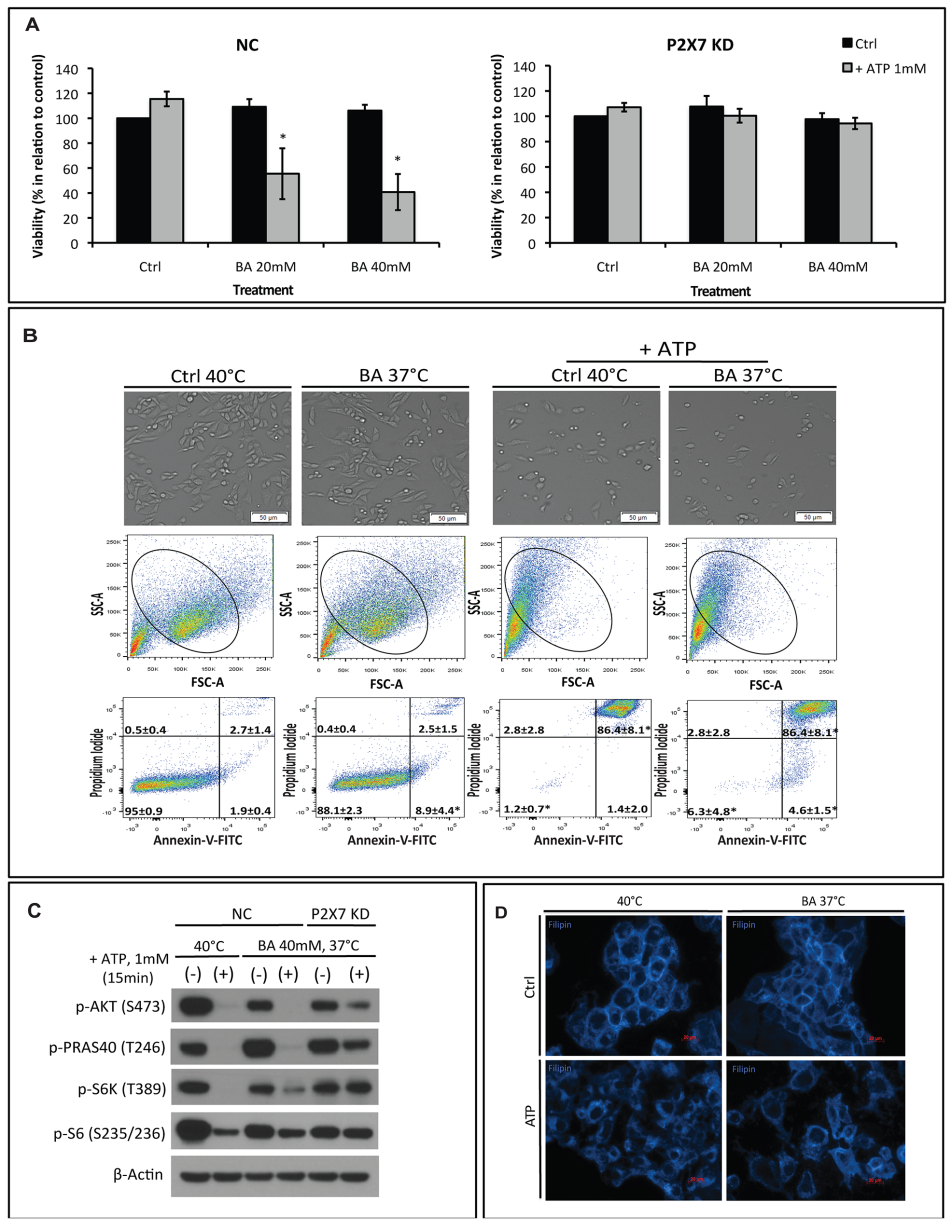
The membrane fluidizer benzyl alcohol (BA) acts similarly as hyperthermia leading to P2X7 hyperactivation at 37°C NC (negative control) or P2X7 KD (P2X7-deficient) cells were exposed to BA alone or together with ATP for 15 min at 37°C (in order to mimic the heat effect per se) or 40°C. Cells treated with media served as control (Ctrl). **(A)** Cell viability and **(B)** images of live cells (upper panel) and FACS analyses of NC cells (middle and bottom panels) were determined. **(C)** Western blot analysis of AKT/PRAS40/mTOR pathway components was performed immediately after treatment. **(D)** Re-organization of cholesterol-rich microdomains in co-treated NC cells was visualized using filipin. *p < 0.05 when compared to control (two-way ANOVA, followed by Bonferroni pos-test, mean ± SD). Bars, 50 μM **(B)** and 20 μM **(D)**.

It is known that, besides lipid raft reorganization, alterations in the nanostructure and physicochemical state of plasma membrane e.g. membrane fluidity may also interfere with protein and receptor functions [12, 13]. We thus replaced hyperthermia with a membrane fluidizer benzyl alcohol (BA) at 37°C in our experimental settings to test if BA treatment could mimic these hyperthermia effects on P2X7 functionality initiated by ATP, as described above. As shown in Figure 4A, in NC cells, BA increased ATP cytotoxicity at 37°C in a dose-dependent manner. This is concurrent with increased cell shrinkage (Figure 4B, upper and middle) and cell apoptosis/necrosis (indicated by percentages of Annexin-V^+^/PI^+^ cells) (Figure 4B, bottom right) as well as blockade of AKT/PRAS40/mTOR signaling (Figure 4C). And such effects were near completely blocked in P2X7 KD cells. Moreover, co-treatment of BA with ATP at 37°C resulted in cholesterol rearrangement in the plasma membrane that is similar to ATP effects at 40°C (Figure 4D). In parallel, neither cholesterol disruption by filipin nor cholesterol loading was able to counteract BA-induced ATP cytotoxicity at 37°C (Supplementary Figure 4).

Taken together, these results imply that enhancements in membrane fluidity caused by hyperthermia augment P2X7 receptor function.

### Hyperthermia and extracellular ATP potentiate chemotherapy cytotoxicity, eliciting maximal cell death

**Figure 5:**
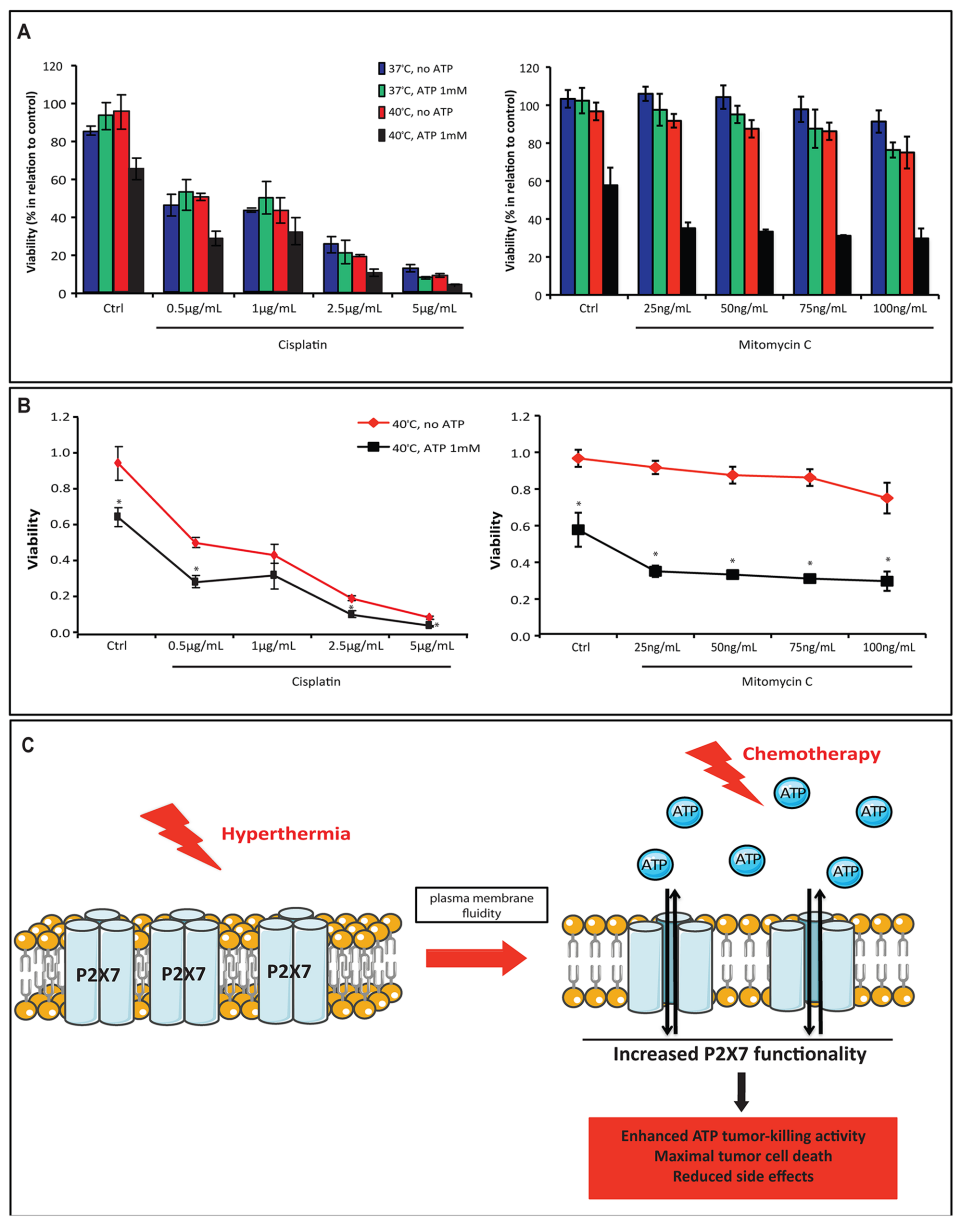
Combination of hyperthermia and ATP with chemotherapy drugs enhances the therapeutic efficacy **(A-B)** MCA38 WT cells were left untreated or treated with cisplatin or mitomycin C at the indicated concentrations overnight prior to ATP pulse treatment (ATP 1 mM, 15 min). Cell viability was evaluated after 24 hr. Overall response **(A)** and drug dose response curve at 40°C **(B)** were analyzed. **(C)** Schematic illustration showing the use of hyperthermia with ATP as a new approach to elicit maximal tumor cell death in association with traditional chemotherapy. Hyperthermia, by promoting plasma membrane fluidity, increases P2X7 sensitivity to ATP and therefore enhances ATP tumor-killing activity. The combination of hyperthermia, ATP and conventional chemotherapy would consequently elicit maximal tumor cell death while circumventing side effects of high dose of cytotoxic drugs. *p < 0.05 when compared cells treated with ATP to cells without ATP treatment (two-way ANOVA, followed by Bonferroni pos-test, mean ± SD).

Next, we investigated the therapeutic potential of combining hyperthermia and ATP with conventional chemotherapeutic drugs (i.e. cisplatin or mitomycin C). Cells were exposed to cisplatin or mitomycin C prior to ATP and/or hyperthermia treatment. We noted that cisplatin induced cancer cell death in a dose-dependent manner, while mitomycin C alone produced a slight cytotoxic effect only at the higher concentration (Figure 5A). Combination of both drugs with hyperthermia and extracellular ATP exhibited additive effects on their cytotoxicity (Figure 5A and 5B). Indeed, this cumulative effect was more pronounced when cells were exposed to lower concentrations of cisplatin and all concentrations of mitomycin C (Figure 5B), suggestive of the potential utility of hyperthermia and ATP in increasing therapeutic efficacy of both drugs while decreasing side effects caused by higher chemotherapy dosage.

Collectively, our data bring a new insight into cancer therapeutics: combination of hyperthermia with ATP or a purine-related drug as an adjunctive therapy to elicit maximal tumor cell death while circum-venting side effects of chemotherapy (as illustrated in Figure 5C).

## DISCUSSION

It has been proposed that high levels of extracellular ATP accumulate within tumor sites, post radiation and chemotherapy [3, 14]. These high levels of extracellular ATP in the tumor microenvironment inhibit tumor growth through the P2X7 receptor, i.e. stimulating antitumor immune responses via P2X7-NLRP3 inflammasome activation [14–16] as well as exerting P2X7-directed cytotoxicity in tumor cells and tumor vascular endothelial cells (thereby limiting angiogenesis) [4, 7, 8, 10, 17–25]. Therefore, maintaining extracellular ATP at high levels in tumor tissues and/or increasing P2X7 functionality might be effective approaches in enhancing the efficacy of traditional cancer therapies.

Local (or superficial) hyperthermia has been used as adjuvant to treat melanoma [26–28]. The clinical use of hyperthermia for cancer either alone or in combination with radiotherapy or chemotherapy has been exploited with mixed effects [28–31]. Improved clinical outcomes have been noted for many types of cancer including prostate [32, 33], breast [34, 35], cervix [36, 37] as well as head and neck cancer [38, 39]. Extracorporeal shock wave therapy (ESWT) is another therapeutic tool utilizing concurrent hyperthermia to treat breast cancer-related lymphedema [40, 41].

Although still controversial, combinations of hyper-thermia with conventional cancer therapies appear to contribute to improved control, cure and/or palliation of certain cancers. Synergistic effects of hyperthermia with chemotherapy are still considered as options in isolated limb perfusion and intraperitoneal chemotherapy for sarcoma and melanoma [28, 42, 43].

Herein, we have identified a novel strategy utilizing hyperthermia that we show to augment ATP-elicited P2X7 functionality thereby maximizing induction of cancer cell death. We have further delineated the underlying molecular mechanisms that could boost effectiveness of chemotherapies such as cisplatin and mitomycin C.

Specifically, we found that hyperthermia effectively sensitizes P2X7 receptor functionality by altering membrane fluidity in response to tumoricidal ATP in MCA38 colon cancer cells. This effect is mediated through the two P2X7 downstream signaling: PI3K/AKT and mTOR that we recently identified [4]. There is growing evidence linking the cellular response to heat stress to changes in lipid composition and architecture of plasma membranes [12]. Nagy et al. [12] showed that either thermal stress or the membrane fluidizer, benzyl alcohol (BA), was able to produce profound alterations in the plasma membrane microdomains and thereby generate stress signal to activate Hsp genes. In this study, we demonstrated that, at 37°C, BA was able to reproduce the hyperthermia effect in potentiating ATP-P2X7 induced cancer cell death, linking increases in membrane fluidity to enhanced P2X7 functionality. Despite hyperthermia and BA being able to activate Hsp gene and increase HSP expression [12, 13, 44], we were unable to observe any HSP modification or interaction with P2X7 in our experimental settings (not shown). This effect was not seen with ethyl alcohol (not shown), which is not considered as a membrane fluidizer. Ethyl alcohol does however have a role in inhibiting NLRP3 inflammasome activation, attenuating IL-1β and caspase-1 cleavage and secretion – pathways related to P2X7 receptor that controls immune system activation [45].

Changes in temperature can also alter solubility, redistribution and activity of lipid raft proteins [46, 47]. Evidences for the involvement of P2X receptors in lipid raft biology are emerging [48, 49] and the presence of P2X7 receptor in this lipid structure has also been described [11, 50, 51]. Accordingly, P2X7 association with lipid rafts varies by cell type. When present in non-raft compartments, P2X7 seems to have increased ion channel activity. In contrast, P2X7 is conferred with a more resistant mode of gating when localized in lipid rafts [11, 50, 51]. In MCA38 cells, P2X7 receptor appears to be present at the non-raft compartments of plasma membrane and its activation occurs independently of cholesterol lipid rafts.

Furthermore, we have demonstrated that hype-rthermia and extracellular ATP have the potential to synergistically increase cytotoxicity of cisplatin and mitomycin C, maximizing tumor cell death. Our data not only reinforce the possible justification of the use of hyperthermia as an adjunctive modality to treat cancer, but also provide a new insight into such treatment option with detailed molecular underpinnings.

On an important note, this strategy would only be applicable for tumor cells expressing the P2X7A receptor splice variant, which is the well-characterized full-length receptor capable of inducing pore formation and apoptosis [52]. Tumors cells expressing truncated/defective P2X7 variant might fail to undergo cell death even with agonist stimulus [53–56].

Taken together, our work highlights the importance of *in vivo* studies to validate the therapeutic potential of hyperthermia, in combination with high levels of ATP or purine-related drugs, to achieve maximal efficacy of more standard cancer treatments.

## MATERIALS AND METHODS

### Reagents and antibodies

Carbenoxolone (CBX), BAPTA-AM and thapsigargin (TG) were purchased from Tocris Bioscience (Ellisville, MO). APT102, the recombinant form of soluble CD39, was kindly provided by Dr. Ridong Chen at APT Therapeutics Inc. (St. Louis, MO). All other chemicals and cell culture media were from Sigma-Aldrich (St. Louis, MO) and other culture reagents from Life Technologies (Carlsbad, CA), unless otherwise stated.

Rabbit anti-mouse P2X7 antibody (#APR-004) was obtained from Alomone labs (Jerusalem, Israel); beta-actin (AC-15, #ab6276) from Abcam (Cambridge, MA); Flotillin-2 (B6, #sc-28320) from Santa Cruz Biotechnology, Inc. (Dallas, TX); Caveolin-1 (7C8, #NB100-615) from Novus Biologicals (Littleton, CO); phospho-AKT (S473) (#9271), phospho-PRAS40 (T246) (#2997), phospho-S6K (T389) (#9205), phospho-S6 (S235/236) (#2211) from Cell Signaling Technology (Danvers, MA); HRP-conjugated goat anti-mouse (#31438), donkey anti-rabbit (#31458) and mouse anti-goat IgG (#31400) and the SuperSignal West Femto Maximum Sensitivity Substrate reagents (#PI-34096) were from Thermo Scientific (Rockford, IL).

### Tumor cell lines

Syngeneic C57BL/6 murine MCA38 colon cancer cells (a gift of Dr. Nicholas P. Restifo, National Cancer Institute) were kindly provided by Dr. Alan B. Frey at New York University School of Medicine [4, 24, 57]. Cells were also tested for Mycoplasma and other infections by mouse IMPACT III PCR Profile via RADIL (Columbia, MO). Cells were maintained in culture flask in RPMI-1640 medium supplemented with 10% fetal bovine serum (FBS), 1% penicillin-streptomycin at 37°C in a 5% CO_2_ atmosphere at 100% humidity. Generation of P2X7 knockdown cell line has been previously established [4]. Selection medium contains 3 μg/ml of puromycin. P2X7-defiecient (P2X7 KD) cells were infected with mouse P2X7 shRNA and negative control (NC) cells were infected with an empty shRNA vector control.

### Hyperthermia treatment

MCA38 cells were pulse-treated with medium or ATP for 15-30 min at 37°C (incubator) or 40°C (water bath) with 5 min pre-incubation. This mild heat temperature was chosen considering the increased cytotoxicity caused by higher temperatures (Supplementary Figure 1). Also, according to Gombos et al [13], at 39.5°C, heat-induced lipid raft destabilization was seen after 10 min of thermal stress and a spontaneous recovery of raft integrity occurred in 45 min. As such, the time course of 15-30 min is adequate to study thermal stress-induced plasma membrane destabilization.

### Antagonist/drugs/plasma membrane disturbance-treatments

Cells were pre-incubated with antagonist carbenoxolone (100 μM), BAPTA-AM (10 μM), thapsigargin (100 nM), and EDTA (0.6 mM) for 30 min, and with soluble CD39 APT102 (2.5 μg/ml) for 15-30 min, before being exposed to pulse treatment with ATP or control medium. Concentration and incubation time for APT102 was based on our previous work [58], which is sufficient to promote total ATP degradation. The cholesterol depleting agents, Methyl-β-cyclodextrin (MCD; 10 mM) or Filipin (10 μM), were incubated for 20 min and removed before ATP treatment. Cholesterol loading at the plasma membrane was obtained by adding water-soluble cholesterol at 100 μg/ml for 30 min before and during ATP exposure. The membrane fluidizer benzyl alcohol (BA), a documented nondenaturant, was added together with ATP for 15 min in order to mimic the hyperthermia effect per se. The chemotherapy drugs Cisplatin and Mitomycin C were added at different concentrations overnight prior to ATP pulse treatment. All compounds were pre-mixed in complete culture medium (RPMI + 10% FBS) at their final concentrations tested before incubation with the cells.

### Cell viability measurements

Cells (7.5 × 10^3^) were seeded into 96-well plates and cultured for 24 hr. Cells were then treated as described above, replaced with fresh culture media, and grown for additional 24 hr. Cells viability was evaluated using Cell Counting Kit-8 (CCK-8, Dojindo Molecular Tech. Inc., Rockville, MD) that measures the activity of cellular dehydrogenases (correlating with cell proliferation), as previously established [24, 59].

### Annexin V and propidium iodide (PI) staining

Phosphatidylserine externalization was determined by the annexin fluorescence signal of an annexin V-fluorescein isothiocyanate conjugate (BioLegend Inc, San Diego, CA) according to the manufacturer’s protocol. Immediately after treatment, cells were trypsinized and centrifuged for 5 min at 1200 rpm, and the supernatant was discarded. Cells were resuspended in Annexin V Binding Buffer at a concentration of 0.25-1.0 × 10^7^ cells/ml and then an aliquot of 100 μl was taken and incubated with 5 μl of FITC-Annexin V and 10 μl of PI for 15 min at room temperature in the dark. Samples were analyzed on BD FACS LSR II cytometer (BD Bioscience, San Diego, CA) using FlowJo V.10 software for analysis (Tree Star Inc, Ashland, OR).

### Analysis of intracellular and extracellular nucleotide levels by high-performance liquid chromatography

Intracellular and extracellular levels of ATP, ADP, and AMP were determined by high performance liquid chromatography (HPLC) as previously described [59–61] with slight modifications. Cells were seeded into 35 mm × 10 mm dishes in culture media and grown for 24 hr until reach 70% of confluence. Cells were then treated with ATP (1 mM) or medium in a final volume of 2 ml for 15 min, at 37°C or 40°C. The supernatant was removed, transferred to a new tube and centrifuged at 2,000 rpm for 10 min at 0°C. A new supernatant aliquot was taken, centrifuged at 5,000 rpm for 5 min at 0°C, precipitated by 5% of 8 M perchloric acid (PCA) and stored at −80°C for subsequent HPLC analyses. The remaining cells were washed with ice-cold HBSS five times to remove excess extracellular ATP. Some wells were lysed with 130 μl of protein lysis buffer for protein concentration measurement; and some wells were harvested with 600 μl of HBSS containing 5% of 8 M PCA and then subjected to three frozen-thaw cycles. Cells were then scraped, transferred to a 1.5 ml Eppendorf tube, pulse sonicated on ice, and then stored at −80°C for subsequent HPLC analysis. 40 μl of each sample were analyzed using a Waters 484 system (Waters Corporation, Milford, MA) as previously described [59–61].

### Ethidium bromide uptake assay

This assay was performed as established previously [4, 62] with slight modifications. Briefly, cells (1.5× 10^4^) were seeded into black 96-well plates. 24 hr later, cells were washed once with HBSS containing Ca^2+^/Mg^2+^ and incubated with 1.27 μM of ethidium bromide (a cell impermeable organic dye) in the absence or presence of ATP (1 mM) for 15 min at 37°C or 40°C, followed by whole cell fluorescence measurement (in arbitrary units of fluorescence, AUF) at 544/610 nm excitation/emission using the SoftMax Pro software on a SpectraMax M5 Microplate Reader (Molecular Devices, Sunnyvale, CA).

### Western blotting

Cells were maintained in 35 mm × 10 mm dishes in culture media for 24 hr until reach 70% of confluence. Cells were treated with freshly made compounds and the reaction was stopped immediately by washing the cells with ice-cold PBS three times. Cells were then lysed in ice-cold modified-RIPA buffer (50 mM Tris-HCl, pH 7.4; 1% NP-40; 0.25% sodium deoxycholate; 150 mM NaCl) supplemented with Protease and Phosphatase Inhibitor Cocktails (Thermo Scientific). The lysates were kept on ice for at least 30 min and then centrifuged at 14,000 rpm for 20 minutes at 4°C. The measurement of protein concentrations and detailed procedures of immunoblotting were described previously [59, 63].

### Immunoprecipitation

Cells were seeded into 100 mm × 20 mm dishes and kept in culture media until reach 80% of confluence. Cells were then treated and the reaction was stopped immediately by washing the cells with ice-cold PBS three times. Cell lysate was obtained as described in Western Blotting section. 150 μg of total cellular protein was incubated with 2 μl of P2X7 or Caveolin-1 antibody or 10 μl of Flotillin-2 antibody with an end-over-end rotation at 4°C for 4 hr. Then, 20 μl of Protein G-Sepharose fast flow (Sigma Aldrich) were added into each mixture and incubated at 4°C on a rotating device overnight. The next day, the mixture were centrifuged at 2500 rpm, for 5 min at 4°C, washed three times with lysis buffer, eluted with 2X SDS reducing sample buffer (Bio-Rad, Hercules, CA) containing 2% of 2-mercaptoethanol and resolved on a 4-12% gradient SDS-page gel (Bio-Rad, Hercules, CA) according to western blot methods.

### Filipin staining

Cells were seeded into 24-well plates at a low confluence and cultured over a cover slip for 3 days until reach 50% of confluence. Then, cells were treated with medium or ATP (1 mM) for 15 min at 37°C or 40°C. The supernatant was removed and cells were fixed with 2% paraformaldehyde (PFA) (Electron Microscopy Sciences, Hatfield, PA). Fixed cells were incubated with filipin (0.5 mg/ml) in PBS for 60 min [64], followed by two washes with PBS and two washes with distilled water. Cell-containing coverslips were then dried, mounted in a microscope glass slide and visualized/analyzed using a Zeiss AxioVert 200M Inverted Fluorescent Microscope (Peabody, MA). Pictures were taken at 40X magnification.

### Statistical analyses

Statistical analyses were performed using Prism 5 (GraphPad, La Jolla, CA). Data are expressed as percentage of control and presented as mean ± SD of at least three independent experiments. Statistical analyses for comparison among multiple groups were performed by one-way analysis of variance (ANOVA), followed by a Tukey post-hoc test. When more than one molecule was mixed to the same well at the same time, a two-way ANOVA was performed, followed by a Bonferroni post-test. Values were considered significant at p < 0.05.

## SUPPLEMENTARY MATERIALS FIGURES



## Abbreviations

ADP: adenosine diphosphate
AMP: adenosine monophosphate
ATP: adenosine triphosphate
APT102: soluble ectonucleotidase CD39
BAPTA-AM: 1,2-Bis(2-aminophenoxy)ethane-N,N,N′,N′-tetra acetic acid tetrakis(acetoxymethyl ester)
BA: benzyl alcohol
CBX: carbenoxolone
eATP: extracellular ATP
EDTA: ethylenediamine tetraacetic acid
HPLC: high performance liquid chromatography
MCD: methyl-β-cyclodextrin
NC: negative control cell line infected with an empty shRNA vector control
P2R: type 2-purinergic receptors
P2X7 KD: P2X7-deficient MCA38 cell line
PCA: perchloric acid
PFA: paraformaldehyde
PI: propidium iodide
TG: thapsigargin
TME: tumor microenvironment
WT: wild type.
