# Correction: Hyperthermia and associated changes in membrane fluidity potentiate P2X7 activation to promote tumor cell death

**DOI:** 10.18632/oncotarget.27641

**Published:** 2022-10-19

**Authors:** Paola de Andrade Mello, Shu Bian, Luiz Eduardo Baggio Savio, Haohai Zhang, Jingping Zhang, Wolfgang Junger, Márcia Rosângela Wink, Guido Lenz, Andréia Buffon, Yan Wu, Simon Christopher Robson

**Affiliations:** ^1^Laboratório de Análises Bioquímicas e Citológicas, Faculdade de Farmácia, Universidade Federal do Rio Grande do Sul (UFRGS), Porto Alegre, RS, Brazil; ^2^Department of Medicine, Beth Israel Deaconess Medical Center, Harvard Medical School, Harvard University, Boston, MA, USA; ^3^Department of Gastroenterology, Tianjin Union Medical Center, Tianjin, P.R. China; ^4^Programa de Imunobiologia, Instituto de Biofísica Carlos Chagas Filho, Universidade Federal do Rio de Janeiro, Rio de Janeiro, RJ, Brazil; ^5^Department of Liver Surgery, Peking Union Medical College Hospital, Chinese Academy of Medical Sciences and Peking Union Medical College, Beijing, P.R. China; ^6^Department of Surgery, Beth Israel Deaconess Medical Center, Harvard Medical School, Harvard University, Boston, MA, USA; ^7^Laboratório de Biologia Celular, Universidade Federal de Ciências da Saúde de Porto Alegre (UFCSPA), Porto Alegre, RS, Brazil; ^8^Departamento de Biofísica e Centro de Biotecnologia, Universidade Federal do Rio Grande do Sul (UFRGS), Porto Alegre, RS, Brazil; ^*^Co-first author; ^#^Joint senior authors

**This article has been corrected:** In Figure 4B, there was an accidental duplication resulting in the mislabeling of both upper gate numbers in the single Annexin-V/PI FACS plot of the “Ctrl 40°C + ATP group”. The incorrectly pasted labels of “2.8 ± 2.8” and “86.4 ± 8.1^*^” were from an adjacent plot. The actual data are “0.6 ± 0.1” and “96.8 ± 3.0^*^, respectively. The corrected Figure 4 is shown below. In addition, the following update has been added to the legends of Figures 1, 3 and 4: ‘Figures 1B and 3C show MCA38 Negative Control (NC), which were subjected to elevated temperature of 40ºC, either in presence or absence of ATP. These same studies were also intentionally shown in Figures 4B and 4D respectively. The figures were assembled and organized in this way to allow direct comparisons of studies, performed in parallel, within each of the treatment groups.’ The authors declare that these corrections do not change the results or conclusions of this paper.

Original article: Oncotarget. 2017; 8:67254–67268. 67254-67268. https://doi.org/10.18632/oncotarget.18595

**Figure 4:**
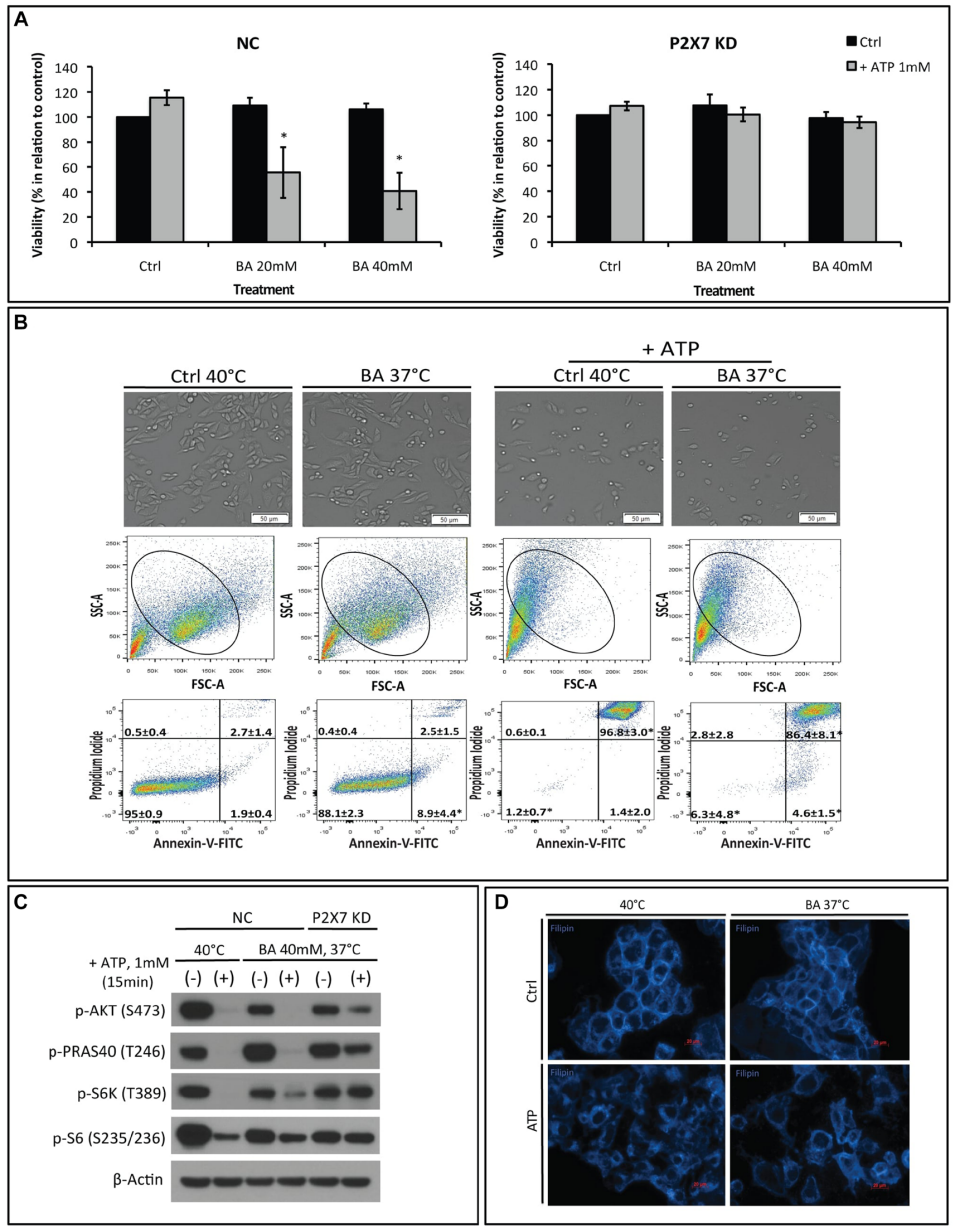
The membrane fluidizer benzyl alcohol (BA) acts similarly as hyperthermia leading to P2X7 hyperactivation at 37°C. NC (negative control) or P2X7 KD (P2X7-deficient) cells were exposed to BA alone or together with ATP for 15 min at 37°C (in order to mimic the heat effect per se) or 40°C. Cells treated with media served as control (Ctrl). (**A**) Cell viability and (**B**) images of live cells (upper panel) and FACS analyses of NC cells (middle and bottom panels) were determined. (**C**) Western blot analysis of AKT/PRAS40/mTOR pathway components was performed immediately after treatment. (**D**) Re-organization of cholesterol-rich microdomains in co-treated NC cells was visualized using filipin. ^*^ *p* < 0.05 when compared to control (two-way ANOVA, followed by Bonferroni pos-test, mean ± SD). Bars, 50 μM (B) and 20 μM (D).

