# Supplementary material for: Hyperthermia and associated changes in membrane fluidity potentiate P2X7 activation to promote tumor cell death
Source: Oncotarget. 2017 Jun 21;8(40):67254–68. doi: 10.18632/oncotarget.18595 (PMC5620171; doi:10.18632/oncotarget.18595)
Supplement: Supplementary file 1 [file oncotarget-08-67254-s001.pdf]

Hyperthermia and associated changes in membrane fluidity potentiate P2X7 activation to promote tumor cell death

SUPPLEMENTARY MATERIALS

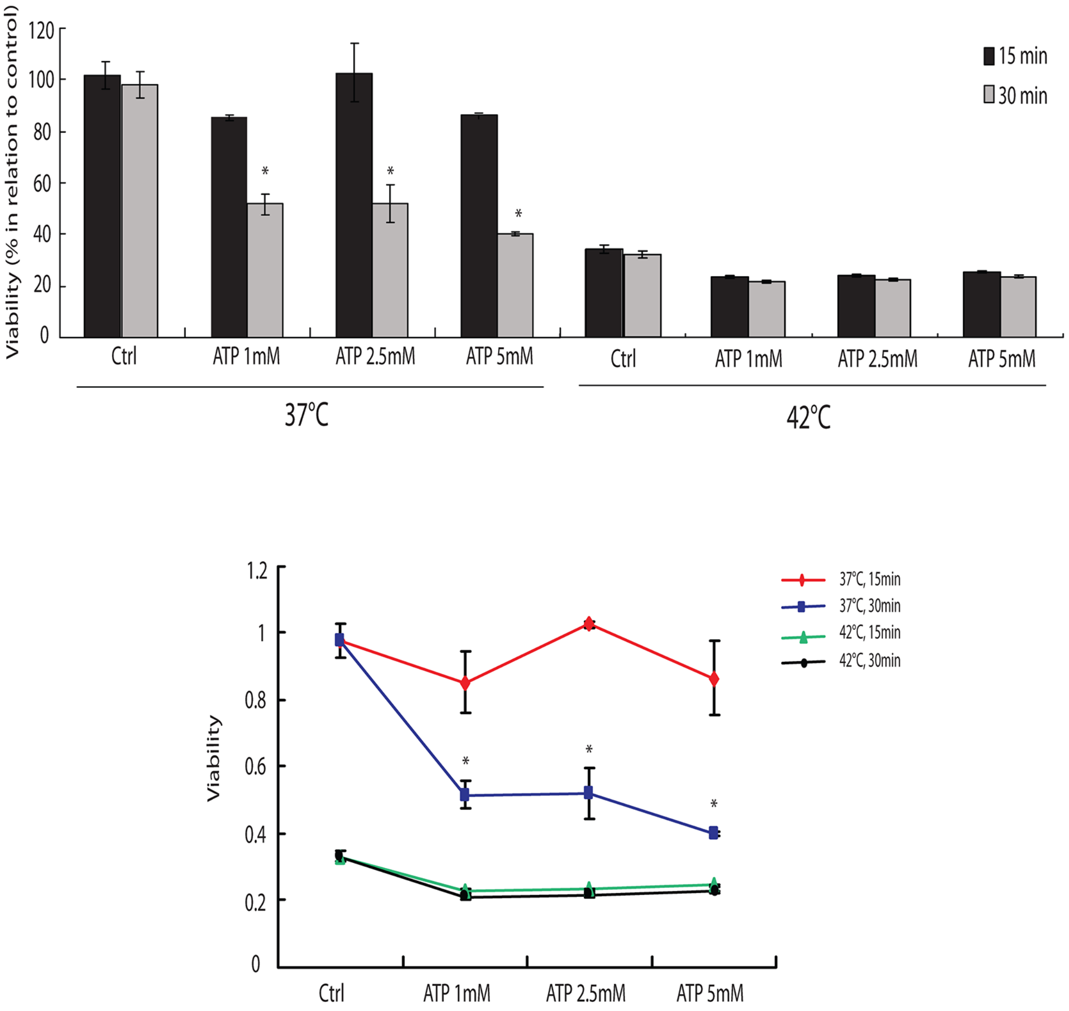

Supplementary Figure 1: Titration of hyperthermia and ATP treatment. MCA38 WT cells were treated with increasing ATP concentrations at 37ºC or 42ºC for 15 or 30 min and cell viability was determined 24 hr later. Note that heat treatment at 42ºC is capable to induce cancer cell death per se. *$p < 0.05$ in relation to the same treatment at 37ºC, 15 min (one-way ANOVA, followed by Tukey pos-test, mean ± SD).

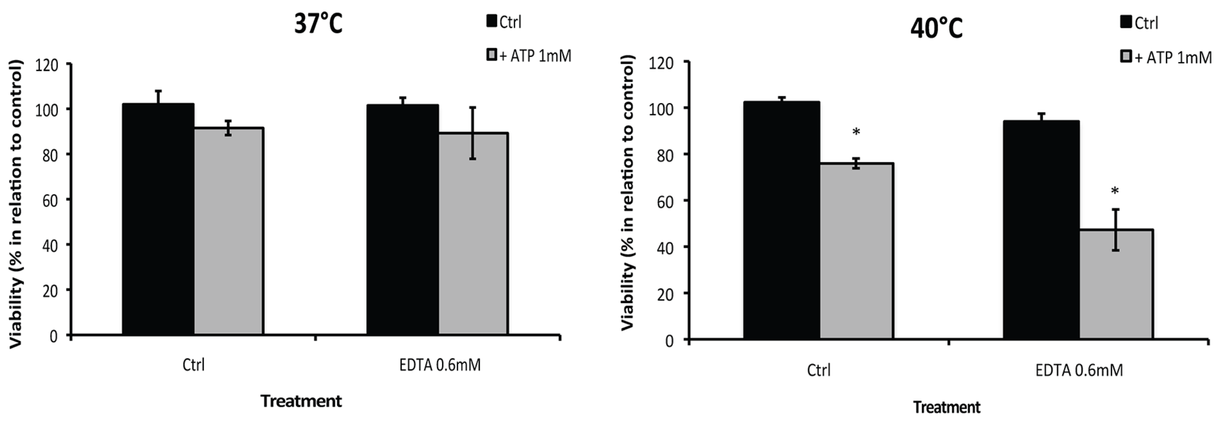

Supplementary Figure 2: Extracellular calcium depletion by EDTA fails to block ATP cytotoxicity at 40ºC. MCA38 WT cells were left untreated or treated with EDTA 0.6mM before being exposed to ATP 1 mM for 15 min at 37ºC or 40ºC. 4 hr later, cell viability was evaluated. *$p<0.05$ as compared to control (two-way ANOVA, followed by Bonferroni pos-test, mean ± SD).

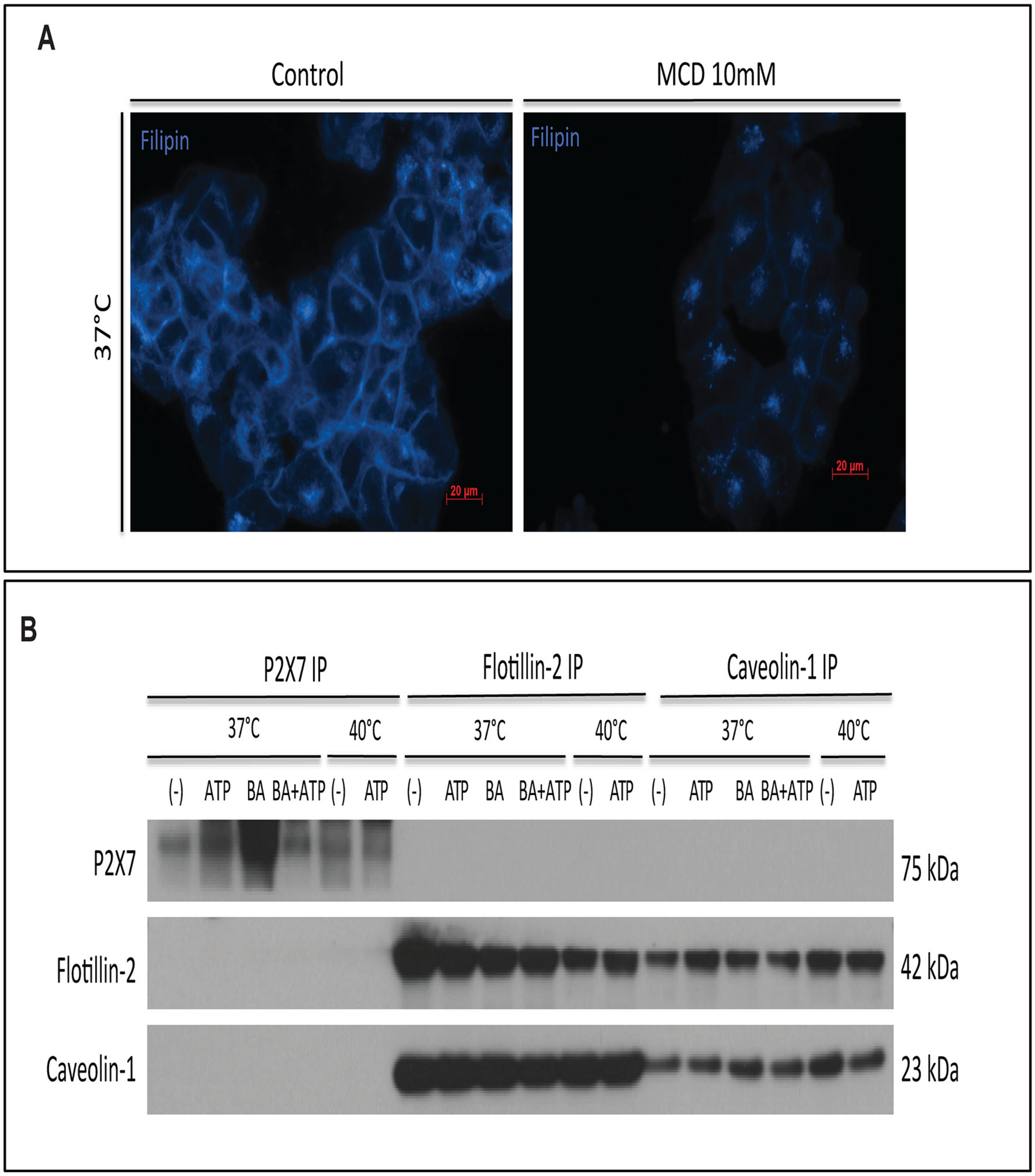

Supplementary Figure 3: Manipulation of plasma membrane cholesterol does not abrogate heat stress or BA induced P2X7 hyperactivation. (A) MCA38 NC cells were left untreated or treated with MCD 10 mM for 20 min followed by cholesterol staining with filipin according to described in experimental procedures. **(B)** Cells were left untreated or treated with ATP, BA or both for 15 min at temperature as indicated. Immunoprecipitation assay was performed to examine physical interactions between P2X7 and lipid raft markers including Caveolin-1 and Flotillin-2. Bars, 20 µM.

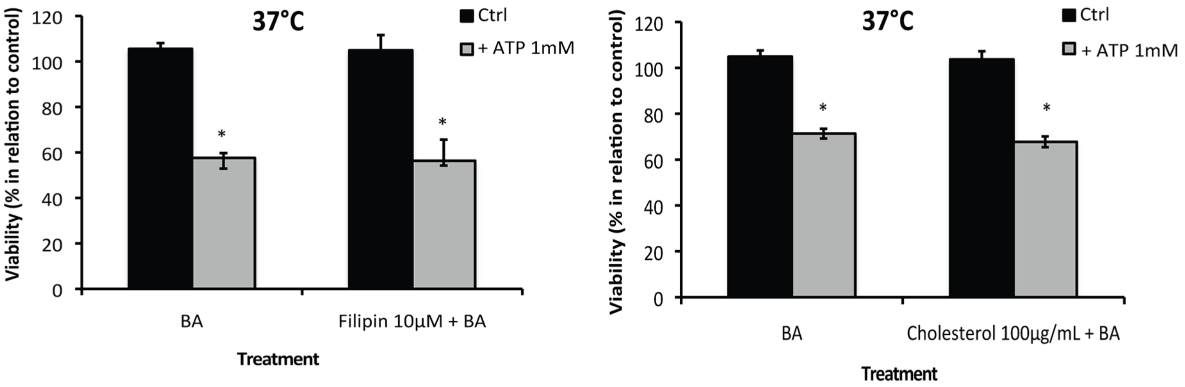

Supplementary Figure 4: Cholesterol disruption by filipin or cholesterol loading at the plasma membrane fail to counteract BA-promoted ATP cytotoxicity at 37ºC. MCA38 NC cells were left untreated or treated with filipin or cholesterol before being exposed to BA alone or together with ATP for 15 min at 37ºC. 24 hr later, cell viability was evaluated. *p<0.05 as compared to control (two-way ANOVA, followed by Bonferroni pos-test, mean ± SD).
